# Investigation of Inter-Limb Symmetry in Knee Extensors Using Different Strength Outcome Measures

**DOI:** 10.3390/diagnostics11101882

**Published:** 2021-10-12

**Authors:** Darjan Smajla, Jure Žitnik, Nejc Šarabon

**Affiliations:** 1 Faculty of Health Sciences, University of Primorska, Polje 42, SI-6310 Izola, Slovenia; darjan.smajla@fvz.upr.si (D.S.); jure.zitnik@fvz.upr.si (J.Ž.); 2 Human Health Department, InnoRenew CoE, Livade 6, SI-6310 Izola, Slovenia; 3 Laboratory for Motor Control and Motor Behaviour, S2P, Science to Practice, Ltd., Tehnološki Park 19, SI-1000 Ljubljana, Slovenia

**Keywords:** knee extension, maximal, submaximal, asymmetry, explosive

## Abstract

Muscle performance between contra-lateral knee extensors is most often assessed using maximal test for isometric/isokinetic torque evaluation. Recently, the rate of force development scaling factor (RFD-SF) has been used to evaluate neuromuscular capacity with a range of submaximal target peak torques, which could highlight other aspects of inter-limb (a)symmetry. The aim of our study was to investigate the differences, associations, and agreement between inter-limb symmetries of knee extensors using maximal torque (T_max_) rate of torque development (RTD), slope of the RFD-SF regression line (k), and theoretical peak of RTD (TP_RTD_). A total of 236 young, healthy athletes participated in the cross-sectional study. All participants performed unilateral knee extension (maximal voluntary contraction protocol and RFD-SF protocol) with both legs in the isometric knee dynamometer. Inter-limb symmetries were calculated for each outcome measure. Our results showed significant differences between all symmetry values (T_max_ (91.7%), RTD (85.2%), k (94.2%), TP_RTD_ (95.9%)). Significant strong correlations were found between symmetry values calculated from k and TP_RTD_ (r = 0.88, *p* < 0.001), while weak correlation was found between T_max_ and RTD (r = 0.17, *p* < 0.01. Fair agreement regarding leg dominance was found between T_max_ and RTD values. Our results suggest that inter-limb (a)symmetries are metric- and task-specific.

## 1. Introduction

Inter-limb (a)symmetry assessment of knee extensors using maximal isometric/isokinetic torque during maximal voluntary contraction is established as a gold standard method for monitoring recovery from an injury or as a guideline before the return to sport [1,2,3]. Regarding the latter, an inter-limb symmetry > 90% has been proposed as a safe criterium when evaluating return to sport [4]. 

The inter-limb (a)symmetries have been most often investigated using the tests that are performed at maximal capacity in isometric [5] and isokinetic conditions [6,7] or using different functional tasks such as jumping [8] and change of direction [9]. Based on this, various outcome measures are used for inter-limb asymmetry calculation, which were already shown as valid and reliable, such as maximal torque (T_max_), maximal rate of torque development (RTD) [8,10], and lately, rate of force development scaling factor (RFD-SF) [11]. In RFD-SF assessment, neuromuscular capacity is evaluated over a wide range of submaximal target peak torques, which could highlight other aspects of inter-limb (a)symmetry (neural stimulation of the muscle, initial motor unit firing rates) [12] in addition to the tests where only maximal torque testing is evaluated. It is not yet known whether the identification of inter-limb (a)symmetries is dependent on the measurement conditions (maximal or submaximal torque testing). To date, only one study compared inter-limb asymmetries between maximal (isometric and isokinetic test) and submaximal conditions (RFD-SF testing) [11]. The authors found a greater prevalence of asymmetry with the submaximal method compared to the maximal method. However, this study used the RFD-SF protocol in which from four to six contractions were performed at each submaximal torque intensity (20–30 pulses overall). We have recently shown that at least 36 pulses should be performed to obtain valid RFD-SF outcome measures [13], while quantification of inter-limb symmetries with RFD-SF outcome measures has been shown to be a reliable method [14].

Therefore, the aim of our study was to investigate the differences between inter-limb symmetries of the knee extensors when calculated from different outcome measures. T_max_ was used as a reference for calculating the inter-limb symmetry, while other symmetry outcome measures were calculated from the RTD during maximal isometric contraction (MVC), k (slope of the RFD-SF regression line), and theoretical peak of RTD (TP_RTD_), where k and TP_RTD_ were calculated from the previously validated RFD-SF protocol [13]. To provide more specific guidelines for inter-limb symmetry testing, we selected outcome measures that evaluate different aspects of muscle capability (T_max_-maximal strength, RTD-rate of torque development, k and TP_RTD_-torque development during submaximal torques). We hypothesised that lower symmetry values from k and TP_RFD_ would be found compared to T_max_ and RTD. Moreover, our aim was to examine the agreement of leg dominance (higher absolute strength values) between T_max_ as a reference outcome measure and other outcome measures. We hypothesised that the agreement of dominance between T_max_ and RTD values would be higher than the agreement between T_max_ and k and T_max_ and TP_RTD_ values in this regard.

## 2. Materials and Methods

### 2.1. Subjects

A total of 236 participants voluntarily participated in the cross-sectional study. Participants were physically active young athletes (Table 1). Inclusion criteria were regular physical activity in the last year with a minimal training frequency of two sessions per week. The preferred leg was determined with the question: “Which leg do you prefer when performing unilateral jumping movements?”. The leg dominance was determined by comparing the absolute values of T_max_ between both legs [15,16]. Informed consent prior to study participation was given by all participants or their parent/guardian (if the participant was under the age of 18). The experiment was approved by Slovenian Medical Ethics Committee (Approval No. 0120-99/2018/5) and was conducted according to the guidelines of the Declaration of Helsinki. 

### 2.2. Study Design

All participants completed a standardised 20-min warm-up routine followed by the tests of unilateral knee extensions (MVC protocol and the RFD-SF protocol) with each of the legs. To maintain consistency with the previous literature, the term RFD-SF is used throughout the paper although torque was measured.

### 2.3. Testing Procedures

**Figure 1:**
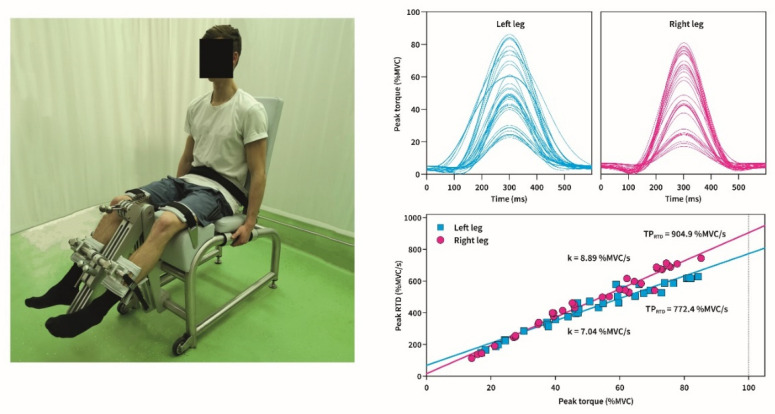
Representative position of the participant in the knee dynamometer (left side); sample recording of torque pulses (individual subject) from the rate of force development scaling factor (RFD-SF) protocol for each leg (top); regression lines (individual subject) depicting the relationship between peak RTD and T_max_ for each leg (bottom); k-slope of regression line (rate of force development scaling factor); TP_RTD_-theoretical peak of RTD.

To determine T_max_ and RTD, participants were seated in an isometric knee dynamometer (S2P, Science to Practice, ltd., Ljubljana, Slovenia) as previously described in Šarabon et al. [17] (Figure 1). A specific warm-up of three submaximal knee extensions at 50, 75, and 90% of participants estimated T_max_ was performed. After a 60 s rest, they performed three explosive MVC’s with each leg. They were instructed to push as fast and hard as possible against the pad positioned on the distal tibia for 3 s, while there was a 60 s rest between each contraction. After RFD-SF familiarization protocol [13], each participant performed at least 15 submaximal explosive contractions at each of the four submaximal levels (20, 40, 60, and 80% of T_max_, 60 pulses in total), aiming to obtain a total of 36 faultless contractions [13] (Figure 1). The target force level was displayed on a computer screen as a horizontal line on a graph. All explosive contractions were cued by an experienced examinator at intervals of approximately 4–5 s.

### 2.4. Data Acquisition and Analysis

The signals from the force transducers were sampled at 1000 Hz by a custom-made LabView 2015 routine (National Instruments Corp., Augustin, TX, USA), low-pass filtered with a 5-Hz cutoff frequency and then analyzed. For each of the three MVCs, a 1 s time interval at highest recorded torque was automatically selected by the software, and the average force during this interval was recorded (T_max_). RTD was quantified as the highest positive value from the first derivative of the torque signal (i.e., the maximal slope of the torque–time curve). The k of RFD-SF was also calculated using a custom-made LabView 2015 routine. Additional information on outcome measures calculation was reported in a separate article [13]. For each subject, T_max_ (Nm) and RTD (Nm/s) were determined from isometric MVC measurements (best of three repetitions), while k (/s) and TP_RTD_ (%MVC/s) were determined from the RFD-SF protocol measurement.

Inter-limb symmetries for all of the main outcome measures were calculated according to the previously published equation [8] (Equation (1)).

(1) $$\mathrm{Symmetry}\;(\%)=100-\left(\frac{\max(\mathrm{preferred}\;\mathrm{or}\;\mathrm{non}-\mathrm{preferred})-\min\;(\mathrm{preferred}\;\mathrm{or}\;\mathrm{non}-\mathrm{preferred})}{\max(\mathrm{preferred}\;\mathrm{or}\;\mathrm{non}-\mathrm{preferred})}\right)\times 100$$

### 2.5. Statistical Analysis

All the statistical analyses were performed using R Statistical Software (version 4.0.5, R Core Team, R Foundation for Statistical Computing, Vienna, Austria). The data distribution in our sample required the use of robust and nonparametric statistical tests for data analysis of inter-limb symmetry. Because preliminary analyses showed negligible to weak correlations among absolute strength outcome measures and inter-limb symmetry values (r = 0.00–0.30), gender pooled data were used for all the analyses presented. Differences between the main outcome measures for inter-limb symmetry (T_max_, RTD, k and TP_RTD_) were examined using robust methods of one-way repeated measures ANOVA (*rmanova* function) and post hoc tests using Hochberg’s correction [18] (*rmmcp* function) from the WRS2 package [19]. The robust alternative to Cohen’s d (δ_R_) was used when reporting the effect size with the following interpretation: negligible (<0.2), small (0.2–0.5), moderate (0.5–0.8), and large (>0.8) [20]. Spearman’s rank correlation test was used to evaluate the relationship between outcome measures. For each participant, leg dominance was determined by comparing the absolute values of T_max_ between both legs. To determine if absolute values of RTD, k, and TP_RTD_ favor the same dominant leg as in T_max_, a kappa coefficient statistic was calculated (with the 95% confidence interval [CI]) and interpreted according to Viera and Garrett [21]. The two-tailed significance level was set at *p* < 0.05.

## 3. Results

**Figure 2:**
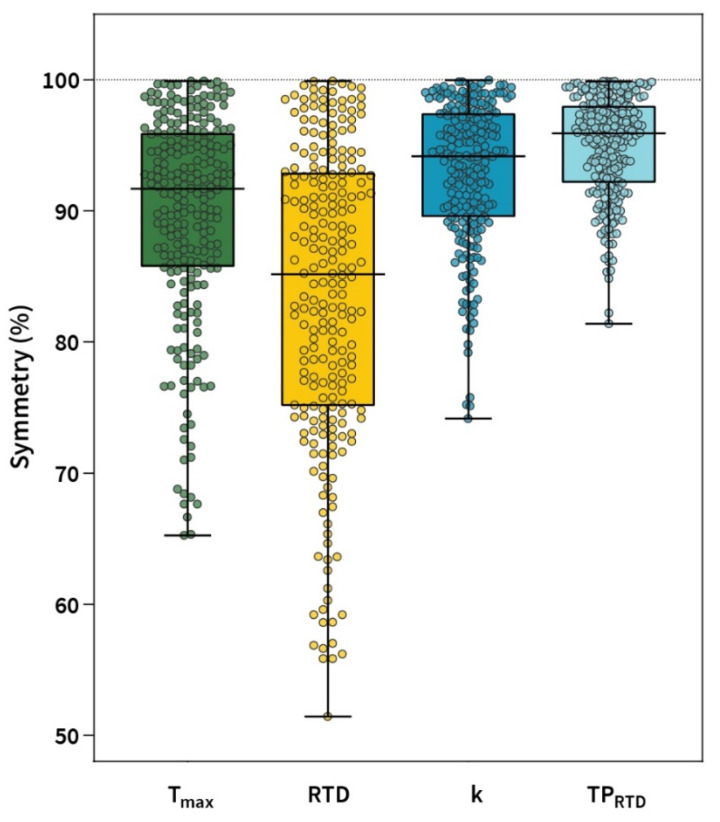
Inter-limb symmetry of knee extensors for different outcome measures. T_max_-maximal isometric torque; RTD-rate of torque development; k-slope of regression line (rate of force development scaling factor); TP_RTD_-theoretical peak of RTD.

The non-parametric ANOVA revealed a significant effect of outcome measure used for inter-limb symmetry calculation (F_1.8, 250.7_ = 78.6, *p* < 0.001). Absolute and inter-limb symmetry values for the main outcome measures are presented in Table 2. Post hoc tests revealed significant differences in inter-limb symmetry values between all the outcome measures (*p* < 0.001 for all paired tests) (Table 3). Individual data of inter-limb symmetries for each outcome measure are presented in Figure 2.

Significant, strong correlations were found between symmetry values calculated from k and TP_RTD_, while a weak correlation was found between T_max_ and RTD (Table 4). No other significant correlations were found. Analysis of leg dominance agreement derived from T_max_ and other outcome measures showed no agreement between T_max_ and k values and T_max_ and TP_RTD_ values, while a fair agreement was found between T_max_ and RTD values (Table 5).

## 4. Discussion

The purpose of the present study was to investigate the leg dominance agreement, differences, and associations agreement between inter-limb symmetries of knee extensors derived from different outcome measures T_max_, RTD, k, and TP_RTD_. The results showed that significant differences exist between the outcome measures. A strong inter-limb symmetry correlation was found between k and TP_RTD_ values, while a weak correlation was found between T_max_ and RTD. The leg dominance agreement analysis showed fair agreement between T_max_ and RTD symmetry values. 

Higher inter-limb symmetry values were found when calculated from TP_RTD_ (95.9%) and k (94.2%), while lower values were calculated from T_max_ (91.7%) and RTD (85.2%). These results refute our hypothesis that inter-limb symmetry values are lower when calculated from k and TP_RRD_. The results of our study are in contrast with the findings of Boccia et al. [11], who observed lower symmetry values of the knee extensors when using k (82.6%) compared to T_max_ (94.2%) and RTD (91.5%) for the calculation of inter-limb symmetry. These differences could be explained by a significantly smaller number of participants included in the aforementioned study (*n* = 22) and the fact that the only group studied were soccer players, in whom rapid submaximal torque production of the knee extensors is more commonly performed with the kicking dominant leg [22]. Moreover, the validity and reliability of the RFD-SF protocol used in their study [11] should be questioned, as we recently reported that at least 36 isometric pulses are required for a reliable and valid calculation of RFD-SF parameters [13]. Furthermore, it is important to note that the RFD-SF outcome measures k and TP_RTD_ are calculated from 36 isometric pulses, which consequently reduces the variability of the results, whereas T_max_ and RTD are derived from single repetitions. In general, we found similar strength-related inter-limb symmetry values compared to previous studies investigating inter-limb symmetries in basketball and volleyball (from 85.74% to 89.6%) [23], volleyball (89.1%) [24], and tennis players (from 84.9% to 95.8%) [9]. However, direct comparisons are limited due to the use of different test protocols. 

The strong significant correlations between k and TP_RTD_ found in our study were expected since TP_RTD_ is derived from the equation TP_RTD_ = k × x + y − int_RTD_, where x = 100%, and y-int_RTD_ is the y axis intercept of the regression line [13]. The observed weak correlation between T_max_ and RTD inter-limb symmetry values was somewhat expected, as other authors have reported mixed results for within-limb correlations between absolute T_max_ and RTD values [25,26,27]. Moreover, several methodological differences exist between the aforementioned studies and our study (RFD calculation, performed task/movement), in addition to the fact that we investigated the relationships between inter-limb symmetry values rather than absolute values. Therefore, no valid comparison with other studies could be made in this regard. The leg dominance agreement between k, TP_RTD_, and RTD absolute values with the most commonly used measure for strength and (a)symmetry evaluation (T_max_) showed fair agreement only between RTD and T_max_, supporting our hypothesis that the leg dominance calculated from RTD would determine the leg with higher absolute values with greater reliability compared to k and TP_RTD_ values. However, it is worth noting that, considering the calculated 95% CI, slight to fair agreement would be a more appropriate term to describe the agreement between T_max_ and RTD. The negative kappa value for k and TP_RTD_ compared to T_max_ indicates random agreement, even when respective 95% CI is considered in the interpretation as the upper bound CI remains negative for both k and TP_RFD_. The lack of significant agreement between T_max_ and RFD-SF measures (k, TP_RTD_) may imply that inter-limb symmetries derived from maximal and submaximal force production need to be more clearly distinguished, as they may represent different modalities. This latter assumption mirrors the assumption of [28] that inter-limb (a)symmetries are metric- and task-specific. Based on that, we suggest strength testing by including both maximal and submaximal (a)symmetry evaluation for in-depth (a)symmetry analysis.

The major limitation of our study is the gender imbalance in the sample and the difference in participants sports activity, which may influence inter-limb symmetry. In further research, a more thorough assessment of inter-limb (a)symmetry using outcome measures from both submaximal (RFD-SF) and maximal strength capacity testing to derive (a)symmetry values should be conducted, followed by in-depth analysis. In future research, the use of different outcome measures to detect inter-limb (a)symmetries should be questioned in more functional movement for greater practical application. 

## Figures and Tables

**Table 1:** Characteristics of participants.

Group	Gender	*n*	Age (Years)	Body Height (cm)	Body Mass (kg)	Left Preferred (*n*)	Right Preferred (*n*)	Training History (Years)
Basketball	Male	69	16.7 ± 1.1	188.0 ± 8.0	79.0 ± 10.7	58	11	7.3 ± 2.2
	Female	38	16.9 ± 1.6	175.3 ± 5.8	70.8 ± 9.6	35	3	6.8 ± 2.5
Students	Male	25	19.7 ± 0.4	182.5 ± 5.7	75.7 ± 8.2	9	16	8.3 ± 3.8
	Female	24	19.7 ± 0.7	166.9 ± 6.0	59.9 ± 7.8	10	14	8.6 ± 4.4
Tennis	Male	50	16.1 ± 3.0	177.1 ± 8.4	67.1 ± 10.1	36	14	8.8 ± 3.8
	Female	30	16.3 ± 2.7	169.2 ± 5.9	61.2 ± 8.0	20	10	8.1 ± 3.7
Overall	Male	144	17.0 ± 2.3	183.2 ± 9.3	74.5 ± 12.0	103	41	8.0 ± 3.2
	Female	92	17.4 ± 2.3	171.1 ± 6.9	65.0 ± 10.1	65	27	7.6 ± 3.4

**Table 2:** Absolute and inter-limb symmetry values (mean ± SD) of knee extensors for different outcome measures.

	Group	Gender	T_max_ (Nm)	RTD (Nm/s)	k (/s)	TP_RTD_ (%MVC/s)
Preferred leg	Basketball	Male	202.1 ± 48.7	1093.7 ± 402.6	9.2 ± 1.0	947.1 ± 71.4
		Female	159.7 ± 39.8	758.4 ± 245.0	8.8 ± 0.9	903.0 ± 71.4
	Students	Male	210.2 ± 48.2	1249.8 ± 311.3	8.9 ± 0.9	921.6 ± 63.4
		Female	142.5 ± 33.2	720.9 ± 232.5	8.6 ± 1.0	889.3 ± 77.5
	Tenis	Male	232.0 ± 75.0	1273.6 ± 477.0	9.1 ± 0.9	936.7 ± 68.1
		Female	197.5 ± 53.2	1022.1 ± 268.1	8.5 ± 0.6	890.3 ± 50.4
	Overall	Male	213.9 ± 60.2	1183.3 ± 422.9	9.1 ± 0.9	939.1 ± 69.1
		Female	167.6 ± 48.1	834.6 ± 280.0	8.6 ± 0.9	895.3 ± 66.6
Non-preferred leg	Basketball	Male	194.6 ± 44.2	1020.1 ± 376.7	9.0 ± 0.9	925.2 ± 72.7
		Female	146.8 ± 35.6	736.3 ± 277.0	8.4 ± 0.9	871.7 ± 68.6
	Students	Male	212.7 ± 45.0	1284.4 ± 343.4	9.1 ± 0.9	934.7 ± 67.9
		Female	144.7 ± 34.9	696.6 ± 238.3	8.5 ± 0.9	884.7 ± 71.7
	Tenis	Male	231.4 ± 78.0	1292.4 ± 504.2	9.1 ± 0.8	938.1 ± 58.9
		Female	191.8 ± 52.2	1059.9 ± 302.9	8.5 ± 0.7	896.5 ± 52.5
	Overall	Male	210.5 ± 60.2	1160.5 ± 438.9	9.1 ± 0.9	931.3 ± 67.2
		Female	160.9 ± 46.5	831.4 ± 317.2	8.5 ± 0.8	883.2 ± 64.8
Symmetry (%)	Basketball	Male	88.6 ± 8.7	82.9 ± 11.9	93.0 ± 5.7	94.7 ± 4.1
		Female	88.6 ± 8.4	80.5 ± 12.6	92.3 ± 5.5	94.6 ± 3.6
	Students	Male	90.5 ± 8.1	90.0 ± 8.1	92.7 ± 5.1	94.9 ± 3.9
		Female	90.4 ± 7.1	81.0 ± 11.0	91.2 ± 5.9	93.8 ± 4.2
	Tenis	Male	89.7 ± 8.0	83.3 ± 12.4	93.2 ± 5.2	95.1 ± 3.8
		Female	92.4 ± 6.0	85.9 ± 7.9	94.7 ± 5.1	96.0 ± 3.6
	Overall	Male	89.3 ± 8.3	84.3 ± 11.7	93.0 ± 5.4	94.9 ± 3.9
		Female	90.3 ± 7.5	82.4 ± 11.0	92.8 ± 5.6	94.8 ± 3.8

T_max_-maximal isometric torque; RTD-rate of torque development; k-slope of regression line (rate of force development scaling factor); TP_RTD_-theoretical peak of RTD.

**Table 3:** ANOVA effect sizes and 95% CI for paired comparisons between symmetry values.

Outcome Measure	T_max_	RTD	k	TP_RTD_
T_max_		0.47 (0.33–0.63) ***	0.34 (0.19–0.47) ***	0.54 (0.39–0.70) ***
RTD			0.66 (0.54–0.83) ***	0.82 (0.68–0.98) ***
k				0.74 (0.60–0.91) ***
TP_RTD_				

T_max_-maximal isometric torque; RTD-rate of torque development; k-slope of regression line (rate of force development scaling factor); TP_RTD_-theoretical peak of RTD; *** *p* < 0.001.

**Table 4:** Result of Spearman’s rank correlation analysis.

Outcome Measure	T_max_	RTD	k	TP_RTD_
T_max_	1	0.17 **	0.06	−0.01
RTD		1	0.03	0.05
k			1	0.88 ***
TP_RTD_				1

T_max_-maximal isometric torque; RTD-rate of torque development; k-slope of regression line (rate of force development scaling factor); TP_RTD_-theoretical peak of RTD. ** *p* < 0.01; *** *p* < 0.001.

**Table 5:** Leg dominance agreement between T_max_ (the reference outcome measure) and other outcome measures.

Outcome Measure	kappa	95% CI
RTD	0.26	0.14–0.38
k	−0.07	−0.20–0.05
TP_RTD_	−0.05	−0.18–0.08

RTD-rate of torque development; k-slope of regression line (rate of force development scaling factor); TP_RTD_-theoretical peak of RTD; 95% CI-95% confidence interval.

## Data Availability

The data presented in this study are available on request from the corresponding author. The data are not publicly available due to privacy reasons.
